# Hormonal and behavioral changes induced by acute and chronic experimental infestation with *Psoroptes cuniculi* in the domestic rabbit *Oryctolagus cuniculus*

**DOI:** 10.1186/1756-3305-6-361

**Published:** 2013-12-19

**Authors:** Claudia Hallal-Calleros, Jorge Morales-Montor, Jaime Abel Vázquez-Montiel, Kurt L Hoffman, Alejandro Nieto-Rodríguez, Fernando Iván Flores-Pérez

**Affiliations:** 1 Facultad de Ciencias Agropecuarias, Universidad Autónoma del Estado de Morelos, Av. Universidad 1001, Col Chamilpa, Cuernavaca CP 62209, México; 2 Departamento de Inmunología, Instituto de Investigaciones Biomédicas, Universidad Nacional Autónoma de México, AP 70228 México DF 04510, México; 3 Centro de Investigación en Reproducción Animal, CINVESTAV-Universidad Autónoma de Tlaxcala, CP 90000, Tlaxcala, México; 4 Facultad de Farmacia, Universidad Autónoma del Estado de Morelos, Av. Universidad 1001, Col Chamilpa, CP 62209, Cuernavaca, Morelos, México

**Keywords:** Animal behavior, Parasite infestation, *Psoroptes*, Mites, Mange, Chinning, Locomotor activity

## Abstract

**Background:**

Parasitic diseases are important in animal production because they cause high economic losses. Affected animals often exhibit stereotypical behavioral alterations such as anorexia and inactivity, among others. Among the diseases that commonly affect domestic rabbits is mange, which is caused by the mite *Psoroptes cuniculi*. Therefore, within the context of the host-parasite relationship, it is critical to understand the mechanisms involved in the alteration of host behavior, in order to better utilize sick animal behavior as a strategy for diagnosis and treatment of disease.

**Methods:**

Rabbits were infested placing mites in the ear conduct. We characterized changes in exploratory behavior and scent marking evoked by acute (1-9 days) and chronic (25-33 days) experimental infestation. Behavior was recorded during ten minutes while the animals were in a 120 cm × 120 cm open field arena divided into 9 squares. Serum cortisol was measured individually using radioimmunoassay kits. Locomotor activity, chinning, rearing and body weight were compared using a Friedman test, the effect of treatment (infested versus non-infested) across time was analyzed using a repeated measures ANOVA, and the Pearson test was used to determine whether chinning and ambulation scores were significantly correlated. Serum cortisol levels and food consumption were analyzed with a Kruskal-Wallis test and body temperature was analyzed with an ANOVA test.

**Results:**

We observed a significant decrease in rearing behavior as early as two days post-infestation, while chinning and locomotor activity were significantly decreased four days post-infestation. Chronic infestation was associated with decreased food intake, significant weight loss, and a trend toward increased serum cortisol levels, while no changes were observed in body temperature.

**Conclusions:**

The presence of visible lesions within the ear canal is commonly used to detect mite infestation in rabbits, but this is possible only after chronic infestation. The behaviors described here may be a useful and economic tool in guiding the early diagnosis of parasitic infestation by *P. cuniculi*, allowing for early treatment and the application of control measures before significant weight loss occurs, thereby avoiding economic losses.

## Background

The domestic rabbit (*Oryctolagus cuniculus*) is an animal species that can be commercialized to several ends, including meat and skin. It has several economic advantages over other domestic species that are reared for human consumption, such as high level of prolificacy, easy husbandry, it does not require grain feeding, and does not compete with humans for food [1]. Moreover, it is also used as an experimental animal model as well as a domestic pet [2]. Parasitic diseases in animals of human consumption are important because they cause high economic losses. Among those parasites that affect rabbits are the ectoparasites that produce mange, which is the most frequently encountered disease in rabbitries. The causative agent of mange is the mite *Psoroptes cuniculi (P. cuniculi)*, which is the most common cause of otitis and skin disease in domestic rabbits, and one of the main reasons for visiting the veterinarian [3,4]. In Mexico, there have been no studies regarding the frequency of mange caused by *P. cuniculi,* nor are there studies that address the economic losses associated with this disease. Mange can cause anorexia, emaciation and death [5], confirming its negative impact on commercial production. Various strategies have been proposed for the treatment of mange caused by *P. cuniculi*, ranging from drugs to the use of entomopathogenic fungi through vaccines [6-8].

When infected by pathogens, including parasites, domestic animals display a series of responses known as acute phase reactions, which include immune, physiological, metabolic and behavioral changes [8]. Thus, it is important to characterize these parameters in order to establish the impact of diseases on animal welfare [9]. In a variety of host-parasite systems, behavioral changes in exploration, general activity, sexual behavior and aggression have been observed in the host. These changes are considered to be adaptive for the parasite, since they facilitate the transmission of parasites between hosts, and/or enhance the probability that parasites are released in an appropriate location [10]. Rabies, Hanta virus and Borna disease, for example, induce aggression and physical contact between individuals, which is thought to increase the likelihood of disease dissemination [10,11]. In trophic transmission, the parasite often modifies the exploratory behavior, activity, aggression, and possibly the sexual attraction of the intermediate host, which makes it more likely to be preyed on by the final host [11]. The behavioral changes induced by infections have been also described for illnesses such as laminitis, metritis, taeniasis, cysticercosis among others, using criteria such as changes in behaviors related to food, drinking water, and locomotor activity [12-14]. In the case of parasitic infestations, it has been proven that parasites can induce alterations in the behavior of some hosts, as has been observed in the mouse model infected with *Trichinella spiralis*[15], *Aspiculuris tetraptera and Syphacia obverlata*[16]. Several studies have suggested that detection of an infection through behavior is advantageous in that it can be carried out at an earlier stage compared to traditional clinical detection [12,13], and being observational, diagnostic costs are practically null. In the case of the rabbit, both males and females exhibit a stereotyped scent marking behavior, called “chinning”, in which the animal rubs the undersurface of its chin on objects within its environment or social group, in order to deposit secretions from the submandibular scent glands [17]. The developmental onset of chinning is closely associated with territoriality and sexual maturity, reflecting the activation and maintenance of this behavior by endogenous testosterone or estradiol secretion in males and females, respectively [18,19]. To our knowledge, this behavior has not been previously associated with infestations. In this work, we analyzed physiological and behavioral changes induced by *P. cuniculi* infestation in rabbits that could be a useful tool to guide the early diagnosis of mange. We found that rearing behavior within an open field arena was the behavior that first showed infestation-associated changes (2 days post-infestation), followed by chinning and locomotor activity, at 4 days post-infestation. Moreover, we observed an early decrease in food consumption correlated with a chronic decrease in body weight, in addition to a tendency toward increased serum cortisol levels in chronically infested animals. This study is a pioneer in addressing potential changes that experimental infestation with *P. cuniculi* can cause on behaviors such as chinning, rearing and locomotor activity in domestic rabbits, and responds to the lack of information regarding this effect that has been cited previously [20].

## Methods

### Animals and experimental groups

All experiments reported here were conducted according to the principles set forth in the Guide for the Care and Use of Laboratory Animals, Institute of Laboratory Animal Resources, National Council, Washington, DC. 1996. This research study was approved by the Ethical Committee of the Veterinary Faculty at UNAM.

Twenty four adult New Zealand female white rabbits were used in these experiments. They were housed individually in wire cages (60 cm L, 90 cm W, 40 cm H) under farm conditions, at room temperature (15-25°C), and fed with Conejina N, Purina® and water *ad libitum*. Rabbits were assigned randomly into three groups, the control group (n = 8), and two infested groups with *P. cuniculi* (n = 8 each). One group was used to represent acute infection, and the other to represent chronic infection.

### Habituation period

In order to allow the rabbits to adapt to the handling conditions and facilities, two weeks prior to infestation with the mite all the management procedures that constitute the study were performed to stabilize the chin marking behavior levels.

### Collection and quantification of the adult mite of *P. cuniculi*

Mites were obtained from the ear of rabbits naturally infested with *P. cuniculi*, infestation was identified by macroscopic lesions and confirmed through a microscope, selecting and counting the mites with mobility and morphology coincident with those described for the mite *P. cuniculi*[21,22].

### Experimental infestation with the adult mite *P. cuniculi*

Sixteen rabbits were infested with 150 mites of *P. cuniculi* placing the mites in the ear conduct, which was occluded with cotton held with tape. The control group underwent the same procedure, excluding the placement of mites [23]. To confirm that rabbits were infested, macroscopic lesions were identified with an otoscope.

### Determination of body weight and body temperature

For the recording of weights we used a digital scale, and the temperature was measured with a digital thermometer placed in the infected ear.

### Food consumption

We provided 400 g of pellet per day for each animal and the next day consumption was determined by subtracting the amount remaining in the feeder.

### Behavioral tests

Behavioral tests were recorded during ten minutes while the animals were in a 120 cm × 120 cm open field arena, made of wire mesh placed on the floor of a room contiguous to that in which the rabbits were housed. The arena was divided into 9 squares (40 cm × 40 cm) by lines painted on the floor. Locomotor activity (ambulation) was quantified as the number of times the rabbit crossed one of these lines during the observation period. Three stacked bricks previously scrubbed with water and a mild detergent, were placed inside the arena, and the number of times the rabbit rubbed its chin against the bricks was quantified [17]. Rearing behavior was quantified as the number of times the rabbit reared up on its hind legs, with its ears erect [24].

### Serum cortisol levels

Serum cortisol was measured individually at the end of the experiment in the three experimental groups as described previously [25]. Briefly, a blood sample was collected by venipuncture at day 33 post infestation and held in an ice bath until centrifugation was achieved to separate serum (1500 rpm for 15 min) and stored at −20°C pending analysis. Serum cortisol concentrations were determined in duplicate using commercial, coated tube radioimmunoassay kits (Pantex, Santa Monica, CA).

### Statistical analysis

Locomotor activity, chinning, rearing and body weight were compared using a Friedman test. The effect of treatment (infested versus non-infested) across time was analyzed using a repeated measures ANOVA, and the Pearson test was used to determine whether chinning and ambulation scores were significantly correlated. Serum cortisol levels and food consumption were analyzed with a Kruskal-Wallis test. Body temperature was analyzed with an ANOVA test.

## Results

**Figure 1:**
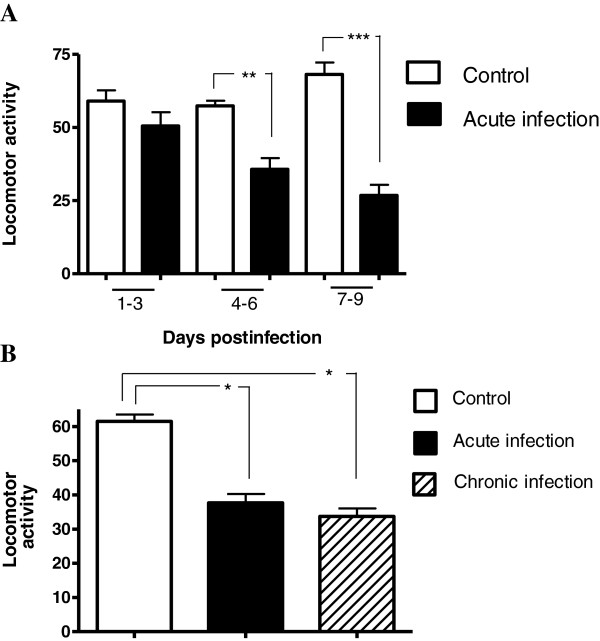
**Locomotor activity diminishes after mite infestation. A)** Locomotor activity during the first 9 days post infestation (acute infestation). **B)** Total locomotor activity during acute and chronic (last 9 days) infestation. Locomotor activity was recorded as the number of times a line within the grid was crossed. The acute infestation data was obtained from the total data from the first 9 days of the acute infestation. Mean ± SE are shown (*P ≤ 0.05, **P ≤ 0.01, ***P ≤ 0.001).

We observed a decrease in locomotor activity in animals infested with *P. cuniculi* during both acute (1–9 days) and chronic (25–33 days) infestation (Figure 1). Figure 1A shows data of ambulatory behavior grouped in 3-day bins. We observed a non-significant decrease in ambulatory behavior in the open field across days 1–3 post-infestation, and statistically significant decreases in this behavior across days 4–6 and 7–9 (P ≤ 0.05), when ambulatory behavior was reduced to approximately 40% of baseline levels. An analysis of total activity revealed that the decrease in locomotor activity was maintained during chronic infestation (Figure 1B).

**Figure 2:**
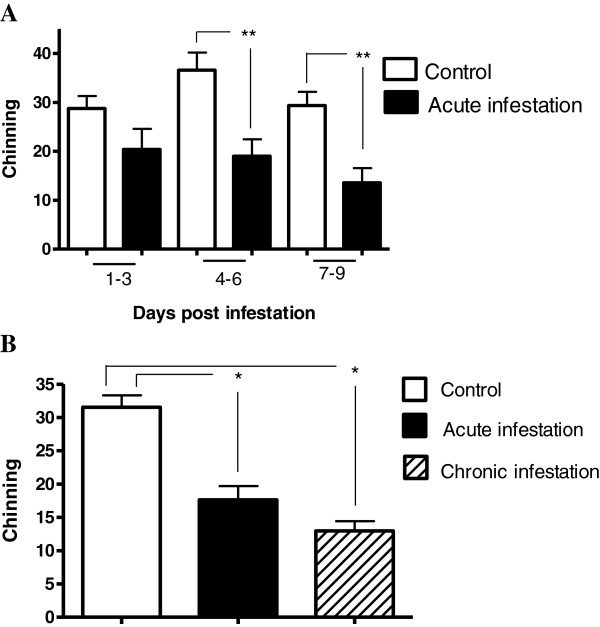
**Chin marking behavior activity. A)** Daily chinning during acute infestation. **B)** Total chinning (total data from first 9 days of acute infestation and last 9 days for chronic infestation). Mean ± SE are shown (*P ≤ 0.05, **P ≤ 0.01).

With regard to chin marking behavior or “chinning”, we observed that the parasitic infestation caused by *P. cuniculi* induced a non-significant decrease in this behavior observable on day one, with a statistically significant decrease beginning on day three post-infestation; this effect was maintained during the 9 days analyzed (Figure 2A). Chinning was further decreased in chronically infested animals, where this behavior was expressed at 40% of baseline levels (Figure 2B). In order to assess whether decreased chinning was secondary to (i.e., dependent on) decreased ambulatory behavior, we used Spearman’s correlation test to determine whether chinning scores were significantly correlated with ambulation scores, on each day post-infestation. We found that, in non-infested rabbits, chinning scores and ambulation scores were correlated only on experimental day 2 (R = 0.898, p = 0.002). By contrast, in infested rabbits, chinning scores were significantly correlated with ambulation scores on days 4, 5, 7, 8, and 9 (day 4, R = 0.71, p = 0.047; day 5, R = 0.731, p = 0.04; day 7, R = 0.871, p = 0.005; day 8, R = 0.776, p = 0.024; day 9, R = 0.719, p = 0.045). This result strongly suggests that decreases in chinning were secondary to decreased ambulatory behavior.

**Figure 3:**
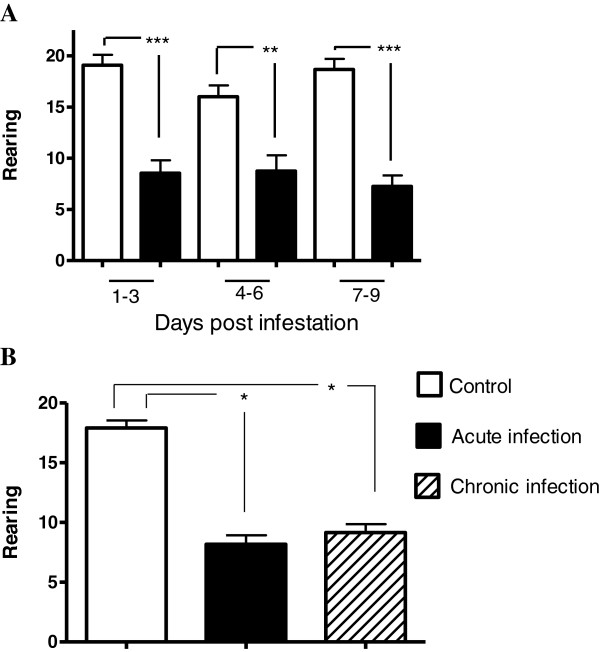
**Rearing behavior. A)** Rearing during acute infestation (first nine days post infestation). **B)** Total rearing during acute and chronic infestation. The acute infestation data was obtained from the total data from the first 9 days of the acute infestation. Mean ± SE are shown (*P ≤ 0.05, **P ≤ 0.01, ***P ≤ 0.001).

Normal behavior of rabbits includes rearing up on their hind legs with their ears erect; in this study, rearing was decreased in the group of rabbits infested with *P. cuniculi* from day two post-infestation, showing a mean decrease of about 50% when comparing control animals to the group of acutely-infested (Figure 3A). Interestingly, across experimental days 1–9, rearing scores were not significantly correlated with ambulation scores in either treatment group, indicating that decreases in rearing behavior in infested rabbits occurred independently of decreases in ambulation.

**Figure 4:**
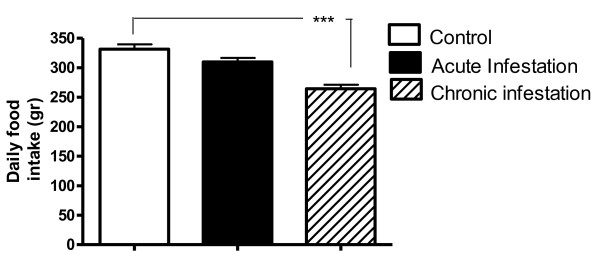
**Voluntary food intake following infestation with *P. cuniculi*.** Food intake was measured during acute (1–9 days) and chronic infestation (25–33 days) and mean ± SD of total values for each experimental group was obtained (Kruskal-Wallis test, Dunas’s multiple comparison test P ≤ 0.001).

The general health status of the rabbits was monitored measuring the temperature, voluntary intake of food and body weight of the animals during the course of the infestation. Total temperature values analyzed during acute infestation with *P. cuniculi* showed no differences when compared between different groups. In the analysis of daily food intake, we observed a decrease in consumption only during chronic infestation, as during acute infestation there are no differences in food intake between infested animals and the control group (Figure 4).

**Figure 5:**
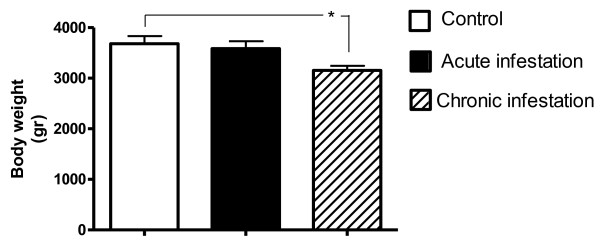
**Body weight.** Body weight was measured during acute (1–9 days) and chronic infestation (25–33 days) and mean ± SD of total values for each experimental group was obtained (ANOVA test, Tukey-Kramer post-test *P ≤ 0.05).

**Figure 6:**
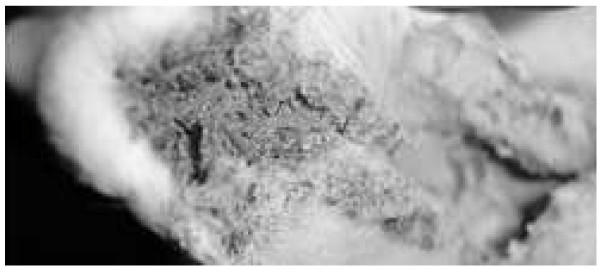
**Mange in rabbit ear chronically infested with *P. cuniculi.***

The decrease in daily voluntary food intake coincided with a decrease in body weight of animals noted during the chronic infestation (Figure 5), correlating with the clinical sign of emaciation that is common in animals infested with mites. At the end of chronic infestation, the ears of rabbits were uncovered and the gross lesions typical of acariasis were corroborated (Figure 6). All the rabbits in the infested group showed mite infestation.

**Figure 7:**
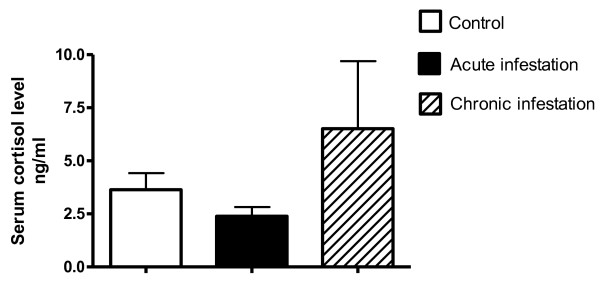
Serum cortisol levels. Serum cortisol levels were measured individually at the end of the chronic infestation (day 33) (Kruskal-Wallis test, mean ± SE is shown).

We analyzed the serum cortisol levels after chronic infestation, observing a clear tendency towards an increase in chronically infested animals. However, inter-individual variability was high, and this difference failed to reach statistical significance (Figure 7).

## Discussion

It has been proposed that parasites such as helminths and arthropods are able to induce modifications in the behavior of their hosts, in order to facilitate their transmission [11,26]. In the present study we observed a decrease in locomotor activity in animals infested with *P. cuniculi* during both acute and chronic infestation. This observation is in agreement with the effect of decreased locomotor activity in rats infested with *Trypanosoma brucei*[27] but also overlaps with reports for *Schistosoma mansoni*, a parasite that, in the hamster, increases locomotor activity at 25 days post infestation (prepatent period), but decreases activity at 40 days post-infestation, in the patent phase [28]. The decrease in locomotor activity in rabbits infested with *P. cuniculi* could be attributed to the inflammation in the ear canal caused by mites.

The parasitic infestation caused by *P. cuniculi* induced a significant decrease in chinning behavior from day three post-infestation and was maintained during chronic infestation. To the best of our knowledge, this is the first study reporting the effect of a parasite on this behavior.

Normal behavior of rabbits includes rearing up on their hind legs with their ears erect; this posture allows rabbits to investigate sights and sounds, and is considered a normal exploratory behavior [29-31]. Here, rearing was decreased in the group of rabbits infested with *P. cuniculi* from day two post-infestation. These results are consistent with the observation that exploratory behavior is reduced in animals parasitized with *Schistosoma mansoni*[27,28], but contrasts with observations of rodents infected with *Toxoplasma gondii*, where exploratory behavior is increased, and the rat shows a diminished fear to the parasite’s definitive host, the cat, thus making the infected rat more susceptible to being predated by the latter [32]. The differences observed among studies can be attributed to the particular parasite involved, each of which would alter host behavior in a way that best promotes its own transmission [33,34]. In the case of the ectoparasite *P. cuniculi*, it does not require that the animal be the victim of a predator in order to continue its life cycle.

*Psoroptes* mites remains on the surface of the skin and, unlike other species, does not penetrate beyond the stratum corneum. However, the mite releases antigenic material on the skin surface, such as saliva and feces, and, using its masticatory organs, causes abrasions of the skin. The combination of mechanical skin abrasion, mite allergen deposition and grooming behaviour by the host in response to the pruritis caused by the mites triggers the subsequent activation of a cutaneous inflammatory response [35]. It has been hypothesized that the production of proinflammatory cytokines causes the activation of the hypothalamic-pituitary-adrenal axis and neurotransmitters that mediate changes in the behavior of animals infected with a pathogen [36]. Cytokines and chemokines have also been reported as modulators of brain development, regeneration, and synapses [37,38]. In the case of infestation with *P. cuniculi*, it is unknown whether there is an association between the observed behavioral changes and the synthesis of antibodies, or with the particular pro-inflammatory cytokine profile induced during acute infestation. This knowledge would establish biological mechanisms responsible for the observed behavioral changes. The rapidity by which *P. cuniculi* infestation induces behavioral changes in the rabbit is remarkable. The possible physiological underpinnings of this response could be similar to those described for sick animal behavior, which is mediated principally by cytokines, and is an adaptive response of the body to resolve the infection. It is known that mites are able to secrete various antigens which act as allergens; in the specific case of *P. cuniculi,* rabbits that are more susceptible to infestation have a weaker response associated with T lymphocytes [6]. There are no reports regarding the cytokines generated in the course of this infestation, however, the pro-inflammatory response induced by the highly related mite *Psoroptes ovis* during sheep scab has been studied. The first approach to investigate the early inflammatory response, was to generate ovine primary keratinocyte cultures challenged with mite derived antigens, and the kinetics of the mRNA response of these cells were monitored by microarray. Authors found that six genes were up-regulated, showing a significant increase in the pro-inflammatory cytokine IL-8 [39]. Afterwards, it was characterized the inflammatory response from sheep skin biopsy samples, achieved through microarrays and qRT-PCR, founding an up-regulation of transcripts for pro-inflammatory cytokine genes IL-1β, IL-6 and IL-8. Furthermore, a sharp computational data analysis demonstrated key roles for TNFα, NF-kB and JUN, all of them being genes playing an important role in the instigation of the pro-inflammatory response, and also for TLR signaling pathway genes (FOS, JUN, JUNB, JUND, ATF3 and MAP2K6), most of them classified as primary response genes for bacterial lipopolysaccharides [40,41]. Concerning the cytokines generated in the course of this infestation, we only can speculate that early changes in the group of acutely infected animals could be due to the action of pro-inflammatory type cytokines, while the behavioral changes observed in chronic infection would be more attributable to the intrinsic characteristics of the parasite and not to the physiological sick animal behavioral response.

With regard to the general health status of the rabbits, we monitored the temperature, voluntary intake of food and body weight of the animals during the course of the infestation. The rabbit’s ears are highly vascularized, and as well as having an auditory function that warns them of the proximity of predators and other external stimuli, they also have an important role in thermoregulation that has been described in detail [42]. It has been reported that stimuli such as heat, fever and dehydration, can modify both the rabbit’s body temperature and the temperature of the skin in the auricular area [43]. However, in this study, total temperature values analyzed during acute infestation with *P. cuniculi* showed no differences.

In the analysis of daily food intake, we observed a decrease in consumption in acute infestation. It has been suggested that food consumption decreases with the aim of creating a hostile environment for the parasite within the host, limiting the reproduction and survival, and this occurs before it affects host survival by the reduction in food intake [44]. There is one previous study in which rabbits were experimentally infected with the internal parasite *T. pisiformis*[14], and which also states that there is a reduction in weight of infected animals, but as they did not determine the consumption of food per day it is difficult to compare with the present study.

Cortisol is an important hormone released in response to stress. Although it acts to restore homeostasis, prolonged secretion of cortisol, which may be due to excessive secretion or chronic stress, leads to major physiological changes, suppressing immune and reproductive functions [45]. We observed a trend towards an increase in serum cortisol levels in chronically infested animals. Serum cortisol is often used in stress and welfare assessments [25,46]. However, it is important to consider that the nature of the aversive stimulus leads to major physiological changes. For example, whereas anxiety is generally believed to cause an increase in levels of glucocorticoid, pain does not reliably result in such an increase [47,48]. Furthermore, differences among individuals, between species and even among breeds should be taken into account. For example, profiles of mean serum cortisol concentration were similar between groups of bulls when a rectal probe was inserted and when a probe was inserted with electrical stimulation applied [49]. With respect to external parasites, serum cortisol levels have been related to tick burden (*Amblyomma americanum*) in cattle. Tick burden also affected various characteristics of growth and metabolism in growing cattle [50], and serum cortisol levels also have been directly associated with intestinal parasite abundance in helminth infestations [44].

## Conclusions

In the sick animal, there are dramatic alterations in behavior, such as increased rest periods and anorexia, among others, which can be mediated by the effects of pro-inflammatory cytokines [51,52]. In the context of host-parasite relationships, it is necessary to understand the adaptive strategies that the parasite uses to facilitate its reproduction, as well as the mechanisms by which the parasite alters host behavior, in order to better interpret sick animal behavior as a strategy in the recovery of health. This study is a pioneer in establishing the behavioral changes that the mite *P. cuniculi* can cause in rabbits, reporting changes as early as two days post-infestation.

Diagnosis of mange is achieved through observation of clinical signs e.g. itching, pruritis and wool loss and ultimately through the detection of mites in skin scrapings. Early stages of infestation are often difficult to diagnose and sub-clinical animals can be a major factor in disease spread [53]. Here, it is proposed that the behaviors assessed may be a useful and economic tool in guiding diagnosis of parasitic infestation by *P. cuniculi*, enabling farmers and veterinarians to detect disease at an early stage, reducing the risk of developing clinical disease and limiting spread. Since the observation of tissue damage is commonly used to detect the presence of the mite in the rabbit, it is important to mention that in the present study, lesions in the experimental infestation with the mite were detected after 21 days post infestation. Moreover, the life cycle of *P. cuniculi* can be studied not only in the domestic rabbit, but also in other animals such as sheep, goats and deer [50], so that the results obtained in this study may be relevant to support future research in other animal species.

## Competing interests

The authors declare that they have no competing interests.

## Authors’ contributions

CHC contributed to conception and design of the study, carried out behavioral experiments, participated in analysis and interpretation of data and drafted the manuscript. JMM participated in the analysis and interpretation of data and in drafting the manuscript. JAVM carried out behavioral experiments and physiological tests. KLF revised the manuscript critically making substantive intellectual contributions and corrected the English version of the manuscript. ANR revised the manuscript critically making substantive intellectual contributions. FIFP participated in the conception and the design of the study, acquisition of funding, general supervision of the research group, analysis and interpretation of data and performed the statistical analysis. All authors read and approved the final manuscript.
